# Down-Regulation of Ca^2+^-Activated K^+^ Channel K_Ca_1.1 in Human Breast Cancer MDA-MB-453 Cells Treated with Vitamin D Receptor Agonists

**DOI:** 10.3390/ijms17122083

**Published:** 2016-12-11

**Authors:** Anowara Khatun, Mayu Fujimoto, Hiroaki Kito, Satomi Niwa, Takayoshi Suzuki, Susumu Ohya

**Affiliations:** 1 Department of Pharmacology, Division of Pathological Sciences, Kyoto Pharmaceutical University, Kyoto 607-8414, Japan; anowarakhatun07@gmail.com (A.K.); ky13291@poppy.kyoto-phu.ac.jp (M.F.); kito@mb.kyoto-phu.ac.jp (H.K.); sniwa@mb.kyoto-phu.ac.jp (S.N.); 2 Graduate School of Medical Science, Kyoto Prefectural University of Medicine, Kyoto 403-8334, Japan; suzukit@koto.kpu-m.ac.jp

**Keywords:** vitamin D receptor, breast cancer, K_Ca_1.1, K^+^ channel, transcription, protein degradation

## Abstract

Vitamin D (VD) reduces the risk of breast cancer and improves disease prognoses. Potential VD analogs are being developed as therapeutic agents for breast cancer treatments. The large-conductance Ca^2+^-activated K^+^ channel K_Ca_1.1 regulates intracellular Ca^2+^ signaling pathways and is associated with high grade tumors and poor prognoses. In the present study, we examined the effects of treatments with VD receptor (VDR) agonists on the expression and activity of K_Ca_1.1 in human breast cancer MDA-MB-453 cells using real-time PCR, Western blotting, flow cytometry, and voltage-sensitive dye imaging. Treatments with VDR agonists for 72 h markedly decreased the expression levels of K_Ca_1.1 transcripts and proteins in MDA-MB-453 cells, resulting in the significant inhibition of depolarization responses induced by paxilline, a specific K_Ca_1.1 blocker. The specific proteasome inhibitor MG132 suppressed VDR agonist-induced decreases in K_Ca_1.1 protein expression. These results suggest that K_Ca_1.1 is a new downstream target of VDR signaling and the down-regulation of K_Ca_1.1 through the transcriptional repression of K_Ca_1.1 and enhancement of K_Ca_1.1 protein degradation contribute, at least partly, to the antiproliferative effects of VDR agonists in breast cancer cells.

## 1. Introduction

Breast cancer is the most common cancer in women, affecting more than two million women worldwide. The multifunctional pro-hormone vitamin D (VD) and its metabolites activate transcriptional factor VR receptor (VDR)-mediated signaling [1]. The active vitamin D metabolite, 1α,25-dihydroxyvitamin D_3_ (calcitriol) regulates cell proliferation and differentiation in cancerous and non-cancerous cells [2,3]. VDR agonists such as calcitriol and its analogs have been shown to exert the potent antiproliferative effects in breast cancer cells [4,5]. Low serum levels of calcitriol are associated with the progression and a high incidence of triple negative breast cancer (TNBC), and VDR-positive breast cancer patients have significantly longer disease-free survival [6]. Recent studies have indicated: (1) a positive correlation between a VD deficiency and the risk of aggressive breast cancer; and (2) the inhibition of migration and invasion by VD analogs [5,7].

Calcitriol modulates transcriptional, post-transcriptional, and post-translational mechanisms in a number of cell types (i.e., pre-mRNA splicing, epigenetic regulation, and protein degradation) [8,9,10]. Calcitriol has also been shown to modulate the transcription by interacting with either the positive or negative VD response element (VDRE) of the promoters of target genes [11]. For example, VDR agonists were previously reported to down-regulate the expression of estrogen receptor (ER) α, which has two potential negative VDREs in the promoter, through a VDR-dependent mechanism in ER-negative breast cancer cells [12,13]. A genome-wide investigation by RNA-Seq technology and The Cancer Genome Atlas identified the transcriptional targets of calcitriol in breast cancer cells [14,15]. Seuter et al. (2013) have demonstrated that calcitriol-induced transcription correlated with the chromatin accessibility of VDR binding regions, and also that calcitriol epigenetically regulated tumor-related VDR target genes through DNA methylation and histone modifications [16,17]. Moreover, calcitriol has been shown to regulate protein degradation through the modulation of proteases and protease inhibitors [10] and specific microRNA (miRNA) processing by enhancing the expression of Dicer in cancer cells [18].

Ca^2+^-activated K^+^ (K_Ca_) channels play important roles in cell proliferation, differentiation, migration, and apoptosis in various cell types by regulating Ca^2+^ signaling. In cancer cells, the dysregulation of K_Ca_ channels is associated with key aspects of proliferation, migration, and invasion during metastasis [19,20,21]. Based on single-channel conductance, K_Ca_ channels have been classified into large-conductance K_Ca_1.1, small-conductance K_Ca_2.x (2.1–2.3), and intermediate-conductance K_Ca_3.1 channels. K_Ca_1.1 is encoded by KCNMA1, and the amplification of KCNMA1 has been correlated with a high tumor stage and poor prognosis in breast cancer [22]. The pharmacological blockade and siRNA-mediated silencing of K_Ca_1.1 induced cell-cycle arrest in the G_0_/G_1_ phase through the down-regulation of cyclin-D1 and cyclin-dependent kinase 4 (CDK4) [21,23] and attenuated breast cancer invasion and metastasis [24]. K_Ca_1.1 transcription and its pre-mRNA splicing are regulated by several hormones. For example, estrogens have been shown to enhance the expression and activity of K_Ca_1.1 via a classic genomic pathway [22,25], and the stress axis-regulated exon (STREX) was inhibited by estrogen and stimulated by progesterone and testosterone [26,27]. Among more than fifty K^+^ channel subtypes, the voltage-gated K^+^ channel, *ether á go-go* 1 (EAG1) K^+^ channel has a negative VDRE in its promoter and is down-regulated by a VDR-dependent pathway in human cervical cancer cells [28,29].

The aim of the present study is to provide new mechanistic insights into the action of VDR agonists in the repression of K_Ca_1.1 transcription and promotion of K_Ca_1.1 protein degradation in breast cancer cells.

## 2. Results

### 2.1. Inhibitory Effects of Calcitriol and Calcipotriol, VDR Agonists, on the Viability of MDA-MB-453 Cells

**Figure 1:**
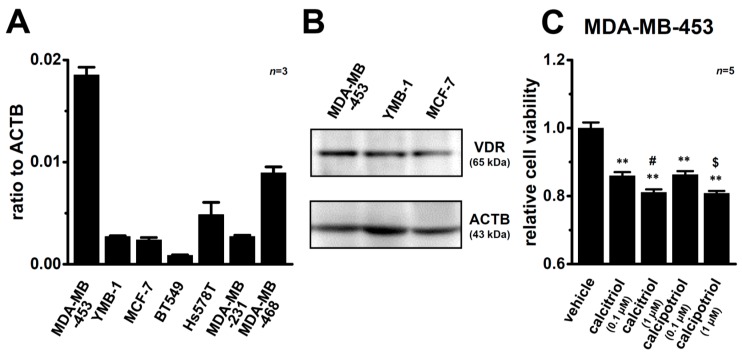
Gene and protein expression of the vitamin D receptor (VDR) in human breast cancer cell lines and effects of treatment with VDR agonists on the viability of MDA-MB-453 cells. (**A**) Real-time PCR assay for VDR in seven human breast cancer cell lines (MDA-MB-453, YMB-1, MCF-7, BT549, Hs578T, MDA-MB-231, and MDA-MB-468) (*n* = 3 for each). Expression levels were expressed as a ratio to β-actin (ACTB); (**B**) Expression of VDR proteins (approximately 65 kDa) in MDA-MB-453, YMB-1, and MCF-7 cells. The molecular weight of VDR was calculated using a prestained protein molecular weight marker (Wako Pure Chemical Industries). Protein lysates of the examined cells were probed by immunoblotting with anti-VDR (D-6) (**upper panel**) and anti-ACTB (**lower panel**) antibodies on the same filter; (**C**) Effects of the treatment with calcitriol or calcipotriol for 72 h on the viability of MDA-MB-453 cells. Cell viability in the vehicle control is arbitrary expressed as 1.0, and data are shown as “relative cell viability” (*n* = 5 for each). Results are expressed as means ± SEM. ** *p* < 0.01 vs. the vehicle control, ^#^, ^$^: *p* < 0.05 vs. calcitriol (0.1 µM) and calcipotriol (0.1 µM) groups, respectively.

We examined the expression levels of VDR transcripts in seven human breast cancer cell lines, MDA-MB-453, YMB-1, MCF-7, BT549, Hs578T, MDA-MB-231, and MDA-MB-468, using a quantitative real-time PCR assay. As shown in Figure 1A, the highest expression of VDR transcripts was detected in MDA-MB-453 cells. Several studies reported VDR-mediated responses in human breast cancer cell lines [4,5]. Concomitant with these findings, similar expression levels of VDR proteins at approximately 65 kDa were observed in the MDA-MB-453, YMB-1, and MCF-7 cells examined in the present study (Figure 1B). Reproducible results are obtained from three independent experiments. As previously reported by Pendás-Franco et al. (2007) [4], the viability of MDA-MB-453 cells was significantly suppressed by the treatment with calcitriol or calcipotriol for 72 h in concentration-dependent manner (Figure 1C).

### 2.2. Inhibitory Effects of the Pharmacological and siRNA-Mediated Blockade of K_Ca_1.1 on the Viability of MDA-MB-453 Cells

**Figure 2:**
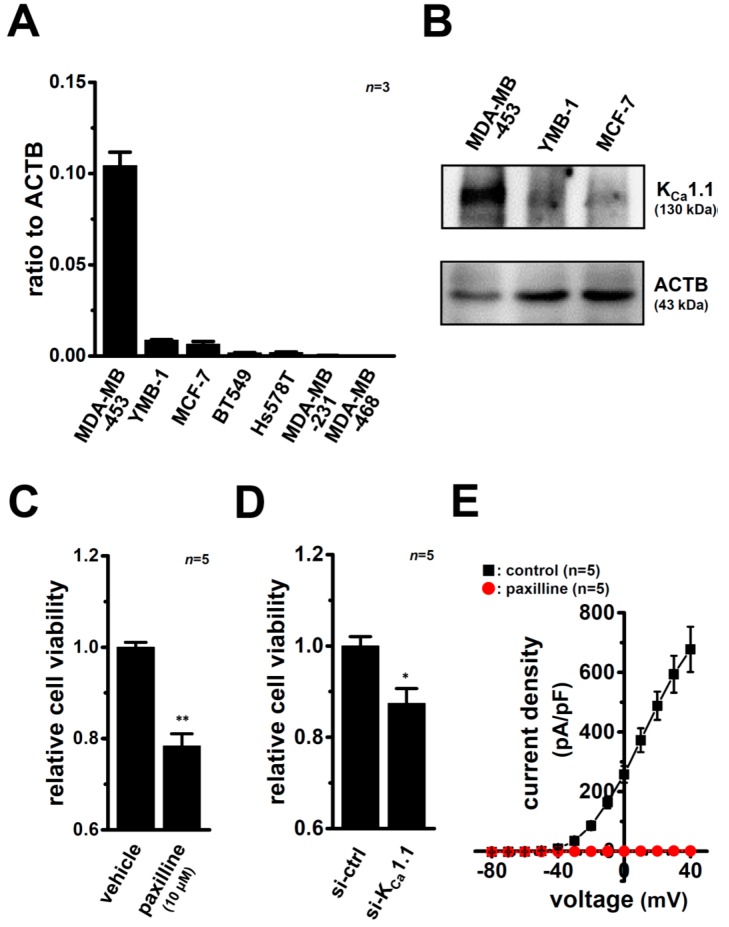
Gene and protein expression of K_Ca_1.1 in human breast cancer cell lines and effects of its pharmacological and/or siRNA-mediated blockade on the viability and K_Ca_1.1 activity in MDA-MB-453 cells. (**A**) Real-time PCR assay for K_Ca_1.1 in seven human breast cancer cell lines (*n* = 3 for each). Expression levels were expressed as a ratio to ACTB; (**B**) Expression of K_Ca_1.1 proteins (about 130 kDa) in MDA-MB-453, YMB-1, and MCF-7 cells. Protein lysates of the examined cells were probed by immunoblotting with anti-K_Ca_1.1 (upper panel) and anti-ACTB (lower panel) antibodies on the same filter; (**C**,**D**) Effects of the treatment with the K_Ca_1.1 blocker, paxilline (10 μM) for 72 h (**C**) and the transfection with K_Ca_1.1 siRNA for 96 h (**D**) on the viability in MDA-MB-453 cells. Cell viability in the vehicle-treated or control siRNA-transfected group is arbitrary expressed as 1.0, and the data are shown as “relative cell viability” (*n* = 5 for each); (**E**) Current-voltage relationship for the current amplitude at the end of the depolarization pulse in MDA-MB-453 cells following treatment with 1 µM paxilline (see Figure S2). Results are expressed as means ± SEM. * *p* < 0.05; ** *p* < 0.01 vs. the vehicle control or control siRNA.

We then investigated the expression levels of K_Ca_1.1 transcripts in seven human breast cancer cell lines using a real-time PCR assay. As shown in Figure 2A, the highest expression of K_Ca_1.1 transcripts was detected in MDA-MB-453 cells. Of the five K_Ca_ channel members examined (K_Ca_1.1/2.1/2.2/2.3/3.1), the expression of K_Ca_1.1 was markedly higher than that of the other members in MDA-MB-453 cells (Figure S1A). Western blotting also revealed higher expression levels of K_Ca_1.1 proteins at approximately 120 kDa in MDA-MB-453 cells than in YMB-1 and MCF-7 cells (Figure 2B). Reproducible results are obtained from three independent experiments. Band signals at 120 kDa disappeared following a preincubation of the primary anti-K_Ca_1.1 antibody with an excess amount of the antigen (Figure S1B). The viability of MDA-MB-453 cells was significantly suppressed by the treatment with the selective K_Ca_1.1 blocker, paxilline (10 µM) for 72 h (Figure 2C) and the transfection of K_Ca_1.1 siRNA for 96 h (Figure 2D). Under the optimum conditions, the expression level of K_Ca_1.1 transcripts was suppressed by approximately 70% in MDA-MB-453 cells (Figure S1C). Furthermore, under whole-cell patch voltage clamp, depolarization of MDA-MB-453 cells evoked outwardly rectifying currents, which are electrophysiological characteristics of voltage-dependent K_Ca_1.1 but not voltage-independent K_Ca_2.x and K_Ca_3.1, and depolarization-induced outward currents were almost completely (over 99%) inhibited by the application of paxilline (1 µM) (*p* < 0.01 at +40 mV) (Figure 2E, Figure S2).

### 2.3. Down-Regulation of K_Ca_1.1 Expression in MDA-MB-453 Cells Treated with VDR Agonists

**Figure 3:**
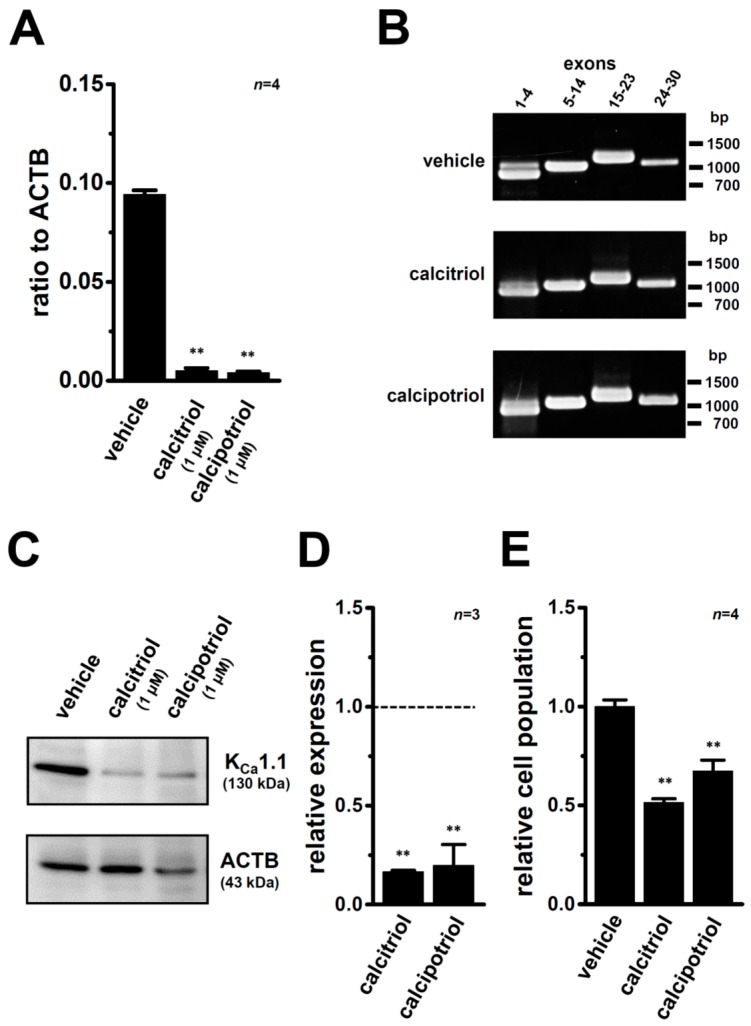
Down-regulation of K_Ca_1.1 transcripts and proteins by the treatment with VDR agonists for 72 h in MDA-MB-453 cells. (**A**) Real-time PCR assay for K_Ca_1.1 in vehicle-, 1 μM calcitriol-, and 1 μM calcipotriol-treated MDA-MB-453 cells (*n* = 4 for each). Expression levels were expressed as a ratio to ACTB; (**B**) Band patterns on agarose gels for the PCR products of K_Ca_1.1 exons (exon 1–4, 5–14, 15–23, and 24–30) in vehicle-, 1 µM calcitriol-, and 1 µM calcipotriol-treated MDA-MB-453 cells. A DNA molecular weight marker is indicated on the right of the gel; (**C**) Protein lysates of vehicle-, 1 µM calcitriol-, and 1 µM calcipotriol-treated MDA-MB-453 cells were probed by immunoblotting with anti-K_Ca_1.1 (upper panel) and anti-ACTB (lower panel) antibodies on the same filter; (**D**) Summarized results are obtained as the optical density of K_Ca_1.1 and ACTB band signals in **C**. After compensation for the optical density of the K_Ca_1.1 protein band signal with that of the ACTB signal, the K_Ca_1.1 signal in the vehicle control was expressed as 1.0 (dotted line, *n* = 3 for each); (**E**) Effects of the treatment with 1 µM calcitriol or 1 µM calcipotriol on the cell surface expression of K_Ca_1.1 proteins by a flow cytometric analysis. Non-permeabilized MDA-MB-453 cells were stained with an Alexa Fluor^@^ 488-conjugated anti-K_Ca_1.1 antibody (extracellular). Data were expressed as the relative cell population of K_Ca_1.1-positive cells to those in the vehicle control (1.0) (*n* = 4 for each). Results are expressed as means ± SEM. ** *p* < 0.01 vs. the vehicle control.

The results of the real-time PCR assay showed that expression levels of K_Ca_1.1 transcripts in MDA-MB-453 cells treated with 1 µM calcitriol or 1 µM calcipotriol for 72 h were more than 90% lower than in the vehicle control (*n* = 4 for each, *p* < 0.01) (Figure 3A). Human K_Ca_1.1 is composed of thirty exons on chromosome 10q22, and more than twenty alternatively spliced variants have been identified in both the N- and C-termini [26]. Non-quantitative RT-PCR examinations were performed using primers specific for exons 1–4 (predicted size of the amplicon: approximately 850 bp), exons 5–14 (approximately 1000 bp), exons 15–23 (approximately 1150 bp), and exons 24–30 (approximately 1060 bp), respectively. As shown in Figure 3B, the band patterns on agarose gels in the calcitriol (middle panel)- and calcipotriol (lower panel)-treated groups were the same as those in the vehicle (upper panel)-treated groups, suggesting that VD analogs do not affect any pre-mRNA splicing processes of K_Ca_1.1 in MDA-MB-453 cells. The protein expression levels of K_Ca_1.1 were subsequently assessed by Western blotting and flow cytometric analyses with different primary anti-K_Ca_1.1 antibodies (see Section 4.3). Similar to the results obtained from the real-time PCR assay, the treatment of MDA-MB-453 cells with VDR agonists resulted in a significant decrease in the expression levels of the K_Ca_1.1 protein (*n* = 4 for each, *p* < 0.01 vs. the vehicle control (relative expression = 1.0, dotted line)) (Figure 3C,D). In the immunocytochemical staining of vehicle- and VDR agonist-treated MDA-MB-453 cells with the Alexa Fluor^@^ 488-conjugated anti-K_Ca_1.1 antibody, which recognized the extracellular region of K_Ca_1.1, the numbers of cells expressing K_Ca_1.1 on the cell surface were analyzed by flow cytometry. The relative cell populations of K_Ca_1.1-positive cells were significantly decreased in MDA-MB-453 cells treated with 1 µM calcitriol or 1 µM calcipotriol (Figure 3E).

### 2.4. Functional Defect in K_Ca_1.1 Activity in MDA-MB-453 Cells Treated with VDR Agonists

**Figure 4:**
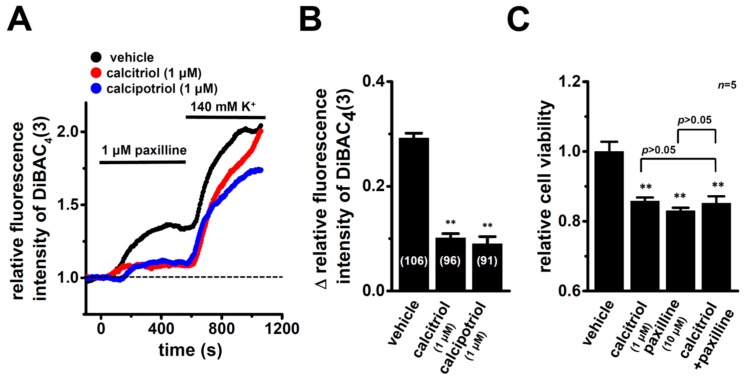
Inhibitory effects of K_Ca_1.1 activities (1 µM paxilline-induced depolarization responses) in MDA-MB-453 cells treated with VDR agonists for 72 h and effects of co-treatment with calcitriol (1 µM) and 1 μM paxilline on the viability of MDA-MB-453 cells. (**A**) Measurement of paxilline-induced depolarization responses in vehicle (black symbol)-, calcitriol (red symbol)-, and calcipotriol (blue symbol)-treated MDA-MB-453 cells. The fluorescence intensity of DiBAC_4_(3) before the application of paxilline at 0 s is expressed as 1.0. The time courses of changes in the relative fluorescence intensity of DiBAC_4_(3) are shown; (**B**) Summarized data are shown as the paxilline-induced ∆ relative fluorescence intensity of DiBAC_4_(3) in vehicle-, calcitriol-, and calcipotriol-treated MDA-MB-453 cells. Cells were obtained from four different batches. Numbers used for the experiments are shown in parentheses; (**C**) Effects of the treatment with calcitriol (1 µM) alone, paxilline (10 µM) alone, and calcitriol plus paxilline for 72 h on the viability in MDA-MB-453 cells. Cell viability in the vehicle-treated is arbitrary expressed as 1.0, and the data are shown as “relative cell viability” (*n* = 5 for each). Results are expressed as means ± SEM. **: *p* < 0.01 vs. the vehicle control.

In order to elucidate the inhibitory effects of the treatment with VDR agonists for 72 h on K_Ca_1.1 activity, depolarization responses induced by paxilline (1 µM), a selective K_Ca_1.1 blocker were measured in MDA-MB-453 cells using the voltage-sensitive fluorescent dye DiBAC_4_(3) imaging. In 100 nM DiBAC_4_(3)-continuously loaded cells (see Section 4.4), K_Ca_1.1 activity is maximal because DiBAC_4_(3) is a potent K_Ca_1.1 activator as shown in our previous study [30]. When fluorescence intensity before the application of paxilline was expressed as 1.0, the increase observed in the relative fluorescence intensity of DiBAC_4_(3) (depolarization response) induced by paxilline was smaller in calcitriol- or calcipotriol-treated MDA-MB-453 cells than in the vehicle control (Figure 4A). Ten minutes after the application of 1 µM paxilline, 140 mM K^+^ solution was applied to cells to omit dead cells and insufficiently DiBAC4(3)-loaded cells, and data were obtained from cells in which large depolarization responses (more than 1.5 in the relative fluorescence intensity of DiBAC_4_(3)) were observed. Summarized data showed that the change in relative fluorescence intensity (Δ relative fluorescence intensity of DiBAC_4_(3)) was significantly lower in calcitriol- or calcipotriol-treated MDA-MB-453 cells than in the vehicle control (Figure 4B). As shown in our previous study, K_Ca_3.1 was functionally expressed in breast cancer YMB-1 cells [31]. In non-treated cells, no depolarization responses were observed by the application of a K_Ca_3.1 blocker, TRAM-34 (1 µM) (not shown). It has been reported that paxilline suppresses Ca^2+^-activated Cl^−^ channel encode by TMEM16A in murine portal vein myocytes [32]. We examined the effects of a selective TMEM16 blocker, T16inh-A01 (1 µM) on the membrane potential of MDA-MB-453 cells, resulting in no depolarization responses by the application of T16inh-A01 (1 µM) (not shown). Furthermore, in order to elucidate whether K_Ca_1.1 is a downstream target of VDR signal, we examined the effects of co-treatment with calcitriol (1 µM) and paxilline (10 µM) for 72 h on the viability of MDA-MB-453 cells. As shown in Figure 4C, no significant differences were observed among calcitriol, paxilline, and calcitriol plus paxilline groups (*p* > 0.05).

### 2.5. Suppression of VDR Agonist-Induced K_Ca_1.1 Protein Degradation by the Potent Proteasome Inhibitor, MG132 in MDA-MB-453 Cells

**Figure 5:**
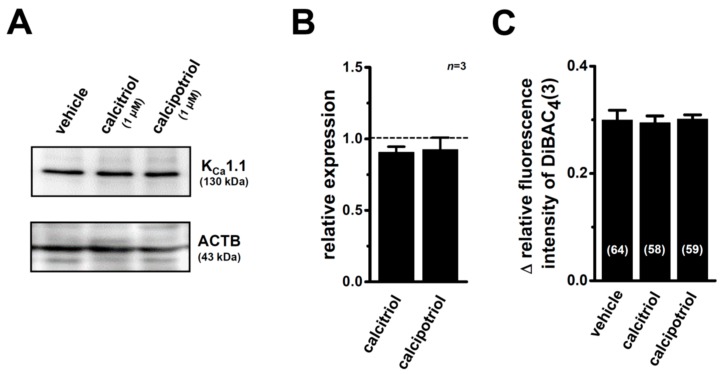
Effects of the proteasome inhibitor, MG132 (100 nM) on VDR agonist-induced K_Ca_1.1 protein degradation and K_Ca_1.1 activity in MDA-MB-453 cells. (**A**) Protein lysates from MDA-MB-453 cells after drug treatments were probed by immunoblotting with anti-K_Ca_1.1 (upper panel) and anti-ACTB (lower panel) antibodies on the same filter; (**B**) Summarized results are obtained as the optical density of K_Ca_1.1 and ACTB band signals in **A**. After compensation for the optical density of the K_Ca_1.1 protein band signal with that of the ACTB signal, the K_Ca_1.1 signal in the vehicle control was expressed as 1.0 (dotted line, *n* = 3 for each); (**C**) Summarized data are shown as the paxilline-induced ∆ relative fluorescence intensity of DiBAC_4_(3) in vehicle-, calcitriol-, and calcipotriol-treated MDA-MB-453 cells. Cells were obtained from three different batches. Numbers used for the experiments are shown in parentheses. Results are expressed as means ± SEM.

As described in Section 1, calcitriol regulates protein degradation through the modulation of proteases and protease inhibitors [10]. In order to elucidate the involvement of the protein degradation process in the down-regulation of the K_Ca_1.1 protein in MDA-MB-453 cells treated with VDR agonists, the effects of MG132 (100 nM) on VDR agonist-induced K_Ca_1.1 protein degradation were examined. Since the treatment of MDA-MB-453 cells with 100 nM MG132 for 48 and 72 h resulted in a marked decrease in the cell viability, MG132 was added 48 h after the treatment with VDR agonists. As shown in Figure 5A,B, the down-regulation of the K_Ca_1.1 protein by the treatment with VDR agonists was almost completely prevented by the treatment with MG132 for 24 h. Similarly, the significant decreases in paxilline-induced depolarization responses in calcitriol- or calcipotriol-treated MDA-MB-453 cells (Figure 4B) were prevented by the treatment with 100 nM MG132 for 24 h (Figure 5C). These results suggest that the VDR signaling pathway plays an important role in K_Ca_1.1 protein degradation processes in breast cancer cells.

### 2.6. Effects of the Treatment with VDR Agonists on Transcriptional Expression Levels of VDR, Androgen Receptor (AR), Estrogen Receptors (ESR1/ERα and ESR2/ERβ), Progesterone Receptor (PGR), and Human Epidermal Growth Factor Receptor 2 (HER2) in MDA-MB-453 Cells

**Figure 6:**
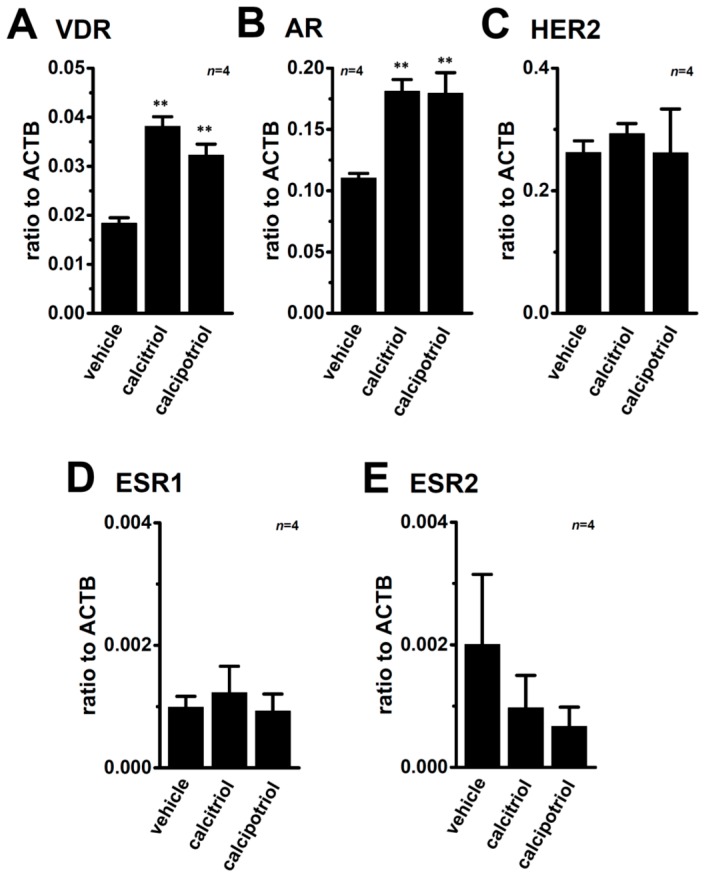
Effects of treatments with VDR agonists on transcriptional expression levels of VDR, androgen receptor (AR), human epidermal growth factor receptor 2 (HER2), and estrogen receptors (ESR1/ERα and ESR2/ERβ) in MDA-MB-453 cells. (**A**–**E**) Real-time PCR assay for: VDR (**A**); AR (**B**); HER2 (**C**); ESR1 (**D**); and ESR2 (**E**) in vehicle-, calcitriol-, and calcipotriol-treated MDA-MB-453 cells (*n* = 4 for each). Expression levels were expressed as a ratio to ACTB. Results are expressed as means ± SEM. ** *p* < 0.01 vs. the vehicle control.

ER, progesterone receptor (PGR), and human epidermal growth factor receptor 2 (HER2) are important tumor markers of breast cancer, and TNBC lacks their expression. Calcitriol and/or its analogs induce the up-regulation of VDR [33] and AR [34] as well as the down-regulation of ERα [12] in prostate and/or breast cancer cells. We examined the effects of the treatment with VDR agonists on the expression levels of their transcripts in MDA-MB-453 cells. Similar to previous studies, the expression levels of VDR (Figure 6A) and AR (Figure 6B) transcripts were significantly increased by the treatment with VDR agonists in MDA-MB-453 cells. On the other hand, no significant changes were observed in the expression levels of HER2 (Figure 6C), ESR1 (Figure 6D), or ESR2 (Figure 6E). The expression levels of PGR transcripts were undetectable in all groups examined.

### 2.7. Contribution of Histone Deacetylase (HDAC) 2 to the VDR Agonist-Induced Down-Regulation of K_Ca_1.1 in MDA-MB-453 Cells

**Figure 7:**
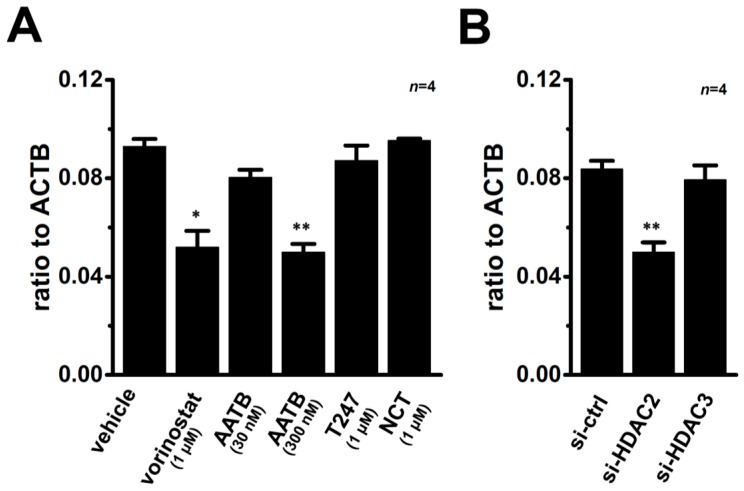
Effects of the pharmacological and siRNA-mediated blockade of HDACs on expression levels of K_Ca_1.1 transcripts in MDA-MB-453 cells. (**A**) Real-time PCR assay for K_Ca_1.1 in MDA-MB-453 cells treated with the following HDAC inhibitors for 48 h (*n* = 4 for each): vorinostat (suberanilohydroxamic acid), a pan-HDAC inhibitor; AATB (4-(acetylamino)-*N*-[2-amino-5-(2-thienyl)phenyl]-benzamide), a HDAC1 (30 nM) and HDAC2 (300 nM) inhibitor; T247 (N-(2-aminophenyl)-4-[1-(2-thiophen-3-ylethyl)-1H-[1], [2], [3]triazol-4-yl]benzamide), a selective HDAC3 inhibitor; and NCT-14b ((*S*)-*S*-7-(adamant-1-ylamino)-6-(tert-butoxycarbonyl)-7-oxoheptyl-2-methylpropanethioate), a selective HDAC6 inhibitor [37]; (**B**) Real-time PCR assay for K_Ca_1.1 in MDA-MB-453 cells transfected with control siRNA (si-ctrl) and siRNAs specific for HDAC2 and HDAC3 (siHDAC2, siHDAC3) for 48 h (*n* = 4 for each). Expression levels were expressed as a ratio to ACTB. Results are expressed as means ± SEM (*n* = 4 for each). * *p* < 0.05; ** *p* < 0.01 vs. the vehicle control or control siRNA-transfected group.

**Figure 8:**
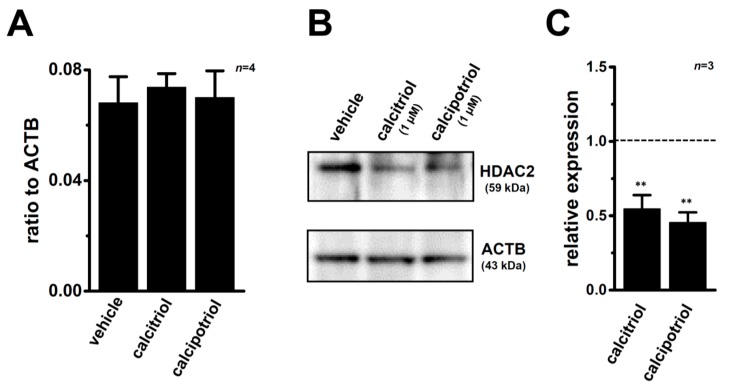
Effects of treatments with VD agonists on expression levels of HDAC2 transcripts and proteins in MDA-MB-453 cells. (**A**) Real-time PCR assay for HDAC2 in VD agonist-treated MDA-MB-453 cells for 72 h (*n* = 4 for each). Expression levels were expressed as a ratio to ACTB; (**B**) Protein lysates of VD agonist-treated MDA-MB-453 cells were probed by immunoblotting with anti-HDAC2 (upper panel) and anti-ACTB (lower panel) antibodies on the same filter; (**C**) Summarized results are obtained as the optical density of HDAC2 and ACTB band signals in **B**. After compensation for the optical density of the K_Ca_1.1 protein band signal with that of the ACTB signal, the HDAC2 signal in the vehicle control was expressed as 1.0 (dotted line, *n* = 4 for each). Results are expressed as means ± SEM (*n* = 4 for each). ** *p* < 0.01 vs. the vehicle control.

Calcitriol may epigenetically regulate the K_Ca_1.1 gene through DNA methylation and histone modifications. As shown in our previous study, the main HDAC members expressed in MDA-MB-453 cells are HDAC1, -2, -3, and -6 [31]. The clinically-available pan-HDAC inhibitor (HDACi) treatment with vorinostat (1 μM) for 48 h significantly suppressed the expression levels of K_Ca_1.1 transcripts (*n* = 4 for each, *p* < 0.05 vs. the vehicle control) (Figure 7A). It has reported that AATB (4-(acetylamino)-*N*-[2-amino-5-(2-thienyl)phenyl]-benzamide) is a selective HDACi for HDAC1 and HDAC2 (half maximal (50%) inhibitory concentration (IC_50_) = 7 and 49 nM for HDAC1 and HDAC2, respectively and IC_50_ ≥ 10 μM for the other HDAC isoforms) [35]. In addition, in our previous study, inhibitory effects of target gene transcription by 30 nM and 300 nM AATB were very similar to those by HDAC1 and HDAC2 siRNAs, respectively [36]. The inhibitory effects of K_Ca_1.1 transcription were demonstrated through the pharmacological blockade of HDAC1 and HDAC2 by 300 nM AATB (*n* = 4, *p* < 0.01), whereas no significant changes were found by the respective inhibition of HDAC1, HDAC3, and HDAC6 by treatments with 30 nM AATB, 1 μM T247 [*N*-(2-aminophenyl)-4-[1-(2-thiophen-3-ylethyl)-1*H*-(1),(2),(3)triazol-4-yl]benzamide], and 1 μM NCT-14b [(*S*)-*S*-7-(adamant-1-ylamino)-6-(tert-butoxycarbonyl)-7-oxoheptyl-2-methylpropanethioate] for 48 h (*n* = 4 for each, *p >* 0.05). Similarly, significant decreases were observed in the expression levels of K_Ca_1.1 transcripts in MDA-MB-453 cells by the siRNA-mediated blockade of HDAC2 (siHDAC2), but not siHDAC3 (*n* = 4 for each, *p* < 0.01 vs. control siRNA, si-ctrl) (Figure 7B). The expression levels of HDAC2 and HDAC3 transcripts were approximately 50% reduced by the respective siRNA transfection (Figure S1D,E). We subsequently examined the effects of treatments with VDR agonists on the expression levels of HDAC2 and HDAC3 transcripts and proteins in MDA-MB-453 cells. No changes were noted in the transcriptional expression levels of HDAC2 in MDA-MB-453 cells treated with the VD agonists (Figure 8A); however, the protein expression level of HDAC2 was significantly decreased by these treatments (Figure 8B,C).

## 3. Discussion

The large-conductance Ca^2+^-activated K^+^ channel, K_Ca_1.1 is associated with high grade and poorly differentiated tumors in breast cancer [22], and is regulated by post-transcriptional and post-translational modifications [38]. VD insufficiency plays an etiological role in cancer, and the active VD metabolite, calcitriol and its potential analogs exert potent antiproliferative effects in breast cancer cells [3,4,5]. However, their genomic effects on ion channels, except for the voltage-gated EAG1 K^+^ channel [28,29], in cancer cells currently remain unclear. The main results of the present study are as follows: (1) the transcriptional repression of K_Ca_1.1 by the treatment of human breast cancer MDA-MB-453 cells with VDR agonists (see Figure 3); and (2) the promotion of K_Ca_1.1 protein degradation by the treatment with VDR agonists in MDA-MB-453 cells (see Figure 5). A VDR agonist, calcitriol largely distributes to serum, and free calcitriol level is more than 100 fold lower than total one [39]. Since the culture medium includes 10% fetal bovine serum, the concentrations of calcitriol applied in this study are much higher than its physiological concentrations.

Calcitriol epigenetically regulates tumor-related genes (i.e., bone morphogenetic protein-2, BMP-2) through DNA methylation and histone modifications [9,15,40]. In our previous study, the pharmacological and siRNA-mediated blockade of HDAC2 or HDAC3 resulted in the down-regulation of the intermediate-conductance Ca^2+^-activated K^+^ channel K_Ca_3.1 in human breast cancer YMB-1 cells [31]. As shown in Figure 7A, the pharmacological blockade of HDAC2 partly but significantly inhibited the expression level of K_Ca_1.1 transcripts in MDA-MB-453 cells, which highly expressed HDAC1, -2, -3, and -6 from the eleven HDAC subtypes [31]. In contrast, the pharmacological blockade of HDAC1, HDAC3, and HDAC6 did not affect expression levels (Figure 7A). In addition, inhibitory rate of cell viability by the treatment with calcitriol (1 μM) was reduced in the presence of a HDAC2 inhibitor, AATB (300 nM) but not a HDAC3 inhibitor, T247 (1 μM) (28.0 ± 1.8, 18.0 ± 1.9 (*p* < 0.05), and 24.3 ± 2.0 (*p* > 0.05) percent in vehicle, AATB, and T247-treated groups, respectively (*n* = 5 for each)). The blocking rates by the inhibition of HDAC were less than 50%, and were markedly smaller than those obtained by the treatments with VDR agonists. The treatments with VDR agonists did not change the expression levels of HDAC2 transcripts (Figure 8A), but significantly suppressed those of HDAC2 proteins (Figure 8B,C). Taken together, VDR agonist-induced defects in the HDAC2 protein expression may be, at least partly, involved in the down-regulation of K_Ca_1.1 in MDA-MB-453 cells.

VDR agonists play a pivotal role in the regulation of pre-mRNA splicing and microRNA (miRNA) processing in cancer cells [41]. In breast cancer cells, VDR agonists regulate the pre-mRNA splicing of the VD target gene and miRNA production [42]. A large number of spliced variants of K_Ca_1.1 with different channel kinetics and surface expression levels are resulted from alternative splicing in its N- and C-termini of it [43,44]. As shown in Figure 3B, VDR agonist-induced modifications in K_Ca_1.1 pre-mRNA splicing were not detected in MDA-MB-453 cells. A class of short non-coding RNA molecules, microRNAs (miRNAs) post-transcriptionally regulate gene expression, resulting in translational repression and gene silencing. Dicer produces mature miRNAs by promoting miRNA processing. The down-regulation of microRNAs mediating the loss of Dicer expression has been associated with breast cancer progression [18,45]. Iosue et al. (2013) reported that calcitriol down-regulated miR-17-5p expression in acute myeloid leukemia cells [46]. A recent study showed that miR-17-5p down-regulated K_Ca_1.1 transcription in malignant pleural mesothelioma [47]. These findings suggest that the transcription of K_Ca_1.1 is enhanced via VDR agonist-induced miR-17-5p down-regulation in breast cancer cells. Conversely, the present study showed that VDR agonists largely suppressed the expression levels of K_Ca_1.1 transcripts in MDA-MB-453 cells (Figure 3A). Therefore, miR-17-5p-mediated post-transcriptional regulation is not responsible for the VDR agonist-induced transcriptional repression of K_Ca_1.1 in MDA-MB-453 cells. It has been reported that several microRNAs are up-regulated by the treatment with calcitriol in cancer cells [48]. Therefore, upregulated microRNAs by VDR agonists may be involved in the transcriptional repression of K_Ca_1.1 in MDA-MB-453 cells.

Previous studies reported the involvement of a functional negative VDRE in the promoter of voltage-gated EAG1 K^+^ channels for its calcitriol-induced down-regulation [28,29]. Since the promoter of K_Ca_1.1 has several estrogen responsive-elements, the transcriptional expression levels of it are up-regulated by estrogens via a classic genomic mechanism [25,49]. Swami et al. (2012) demonstrated that calcitriol repressed the ER promoter in human breast cancer cells via two negative VDREs [12]. However, the expression levels of ESR1 and ESR2 transcripts were originally very low in MDA-MB-453, and no changes were induced by the treatment with VDR agonists (Figure 6D,E). These results suggest that the modification of estrogen production and ER expression may not be responsible for the VDR agonist-induced transcriptional regulation of K_Ca_1.1 in breast cancer cells.

As shown in Figure 5, the proteasome inhibitor almost suppressed the VDR agonist-induced down-regulation of K_Ca_1.1 in MDA-MB-453 cells. The NEDD4 family of E3 ubiquitin ligases (NEDD4-1/NEDD4-2) have been shown to regulate several K^+^ channels such as K_V_1.3 and HERG [37,50,51]. In the present study, no significant changes in the expression levels of NEDD4-1 and NEDD4-2 transcripts were found in MDA-MB-453 cells treated with VDR agonists (Figure S3A,B). Alvarez-Díaz et al. (2010) previously reported that calcitriol regulates protein degradation through the modulation of cysteine protease and matrix metalloproteases (MMP-2 and MMP-9) [10]. To date, there have been no studies on the modification of E3 ubiquitin ligases by VD analogs. K_Ca_1.1-interacting partners were recently summarized by Kim and Oh (2016) [52]. The formation of K_Ca_1.1 complexes has been suggested to prevent the protein degradation of K_Ca_1.1, whereas the down-regulation of K_Ca_1.1 partner molecules by VDR agonists promotes it. Further studies will be needed in order to clarify the mechanisms underlying the protein degradation of K_Ca_1.1 via VDR signaling pathways in breast cancer cells.

Zhao and Feldman (2001) found that calcitriol enhanced AR transcriptional activity in androgen-sensitive LNCaP prostate cancer [53]. AR is also an important therapeutic target of TNBC, and non-steroidal anti-androgens are currently being investigated in AR-positive TNBC [54]. Recent studies showed that calcitriol promotes the transcription of AR in TNBC [13,55]. In the present study, the VDR agonist-induced up-regulation of AR was detected in AR-positive, ER- and PGR-negative MDA-MB-453 cells (Figure 6A). These results suggest that combination therapy with vitamin D analogs and anti-androgens has potential for the treatment of TNBC.

## 4. Materials and Methods

### 4.1. Cell Culture and Cell Viability Assay

The breast cancer cell lines MDA-MB-453, YMB-1, MCF-7, Hs578T-Luc and BT-549 were supplied by the RIKEN BioResource Center (RIKEN BRC, Tsukuba, Japan) and Health Science Research Resources Bank (HSRRB, Osaka, Japan). MDA-MB-231 and MDA-MB-468 were supplied by Nishiguchi (Kyoto Pharmaceutical University, Kyoto, Japan). They were maintained at 37 °C in 5% CO_2_ with RPMI 1640 medium, Dulbecco’s modified Eagle’s (DMEM) medium, or Leibovitz’s L-15 medium containing 10% fetal bovine serum (Sigma, St. Louis, MO, USA) and a penicillin (100 units/mL)-streptomycin (0.1 mg/mL) mixture [36]. A cell viability assay using WST-1 (2-(4-Iodophenyl)-3-(4-nitrophenyl)-5-(2,4-disulfophenyl)-2*H*-tetrazolium, monosodium salt), which is a colorimetric assay to measure the viable cell numbers by the cleavage of tetrazolium salts was performed according to our previous study [36]. Briefly, using a density of 10^5^ cells/mL, cells were cultured in duplicate in 96-well plates for 0–4 days. Four hours after the addition of WST-1 reagent (Dojindo, Kumamoto, Japan) into each well, the absorbance was measured in a microplate reader Multiskan FC (Thermo Fisher Scientific, Yokohama, Japan) at a test wavelength of 450 nm as a reference wavelength of 620 nm. In the siRNA-mediated blockade of K_Ca_1.1, HDAC2, and HDAC3, Lipofectamine^®^ RNAiMAX reagent (Thermo Fisher Scientific) was used [36]. Commercially available siRNA oligonucleotides against human K_Ca_1.1/HDAC2/HDAC3 and control siRNA (type A) were purchased from Santa Cruz Biotechnology. The expression levels of the target transcripts were assessed 48 h after the transfection of siRNAs using a real-time PCR assay, and cell viability was measured using the WST-1 assay. Cell culture medium, calcitriol, calcipotriol, paxilline, and other chemicals were obtained from Sigma-Aldrich (St. Louis, MO, USA) or Wako Pure Chemical Industries (Tokyo, Japan).

### 4.2. RNA Extraction, Reverse Transcription, and Real-Time PCR

Total RNA extraction from cell lines and reverse-transcription were performed as previously reported [36]. cDNA products were amplified with gene-specific PCR primers, designated using Primer Express^TM^ software (Ver 3.0.1, Life Technologies, Carlsbad, CA, USA). Quantitative, real-time PCR was performed using SYBR Green chemistry on an ABI 7500 sequence detector system (Applied Biosystems, Foster City, CA, USA). The following gene-specific PCR primers of human origin were used for real-time PCR: K_Ca_1.1 (GenBank accession number: NM_001014797), 1120–1239, amplicon = 120 bp; K_Ca_2.1 (NM_002248), 649–764, 116 bp; K_Ca_2.2 (NM_021614), 1492–1612, 121 bp; K_Ca_2.3 (NM_002249), 2042–2146, 105 bp; K_Ca_3.1 (NM_002250), 1475–1595, 121 bp; HDAC2 (NM_001527), 298–405, 108 bp; HDAC3 (NM_003883), 699–819, 121 bp; VDR (NM_000376), 1034–1153, 120 bp; AR (M20132), 2457–2583, 127 bp; estrogen receptor α (ERα/ESR1) (NM_000125), 709–828, 120 bp; ERβ/ESR2 (NM_001437), 610–729, 120 bp; HER2/ERBB2 (NM_004448), 1440–1559, 120 bp; progesterone receptor (PGR) (M15716), 1924–2043, 120 bp; NEDD4-1 (NM_006154), 1372–1491, 120 bp; NEDD4-2 (AY312514), 1039–1158, 120 bp; β-actin (ACTB) (NM_001101, 411–511), 101 bp. Unknown quantities relative to the standard curve for a particular set of primers were calculated as previously reported [36], yielding the transcriptional quantitation of gene products relative to the endogenous standard, ACTB.

In order to examine whether the pre-mRNA splicing of K_Ca_1.1 is changed by a treatment with VDR agonists, the PCR amplification of partial fragments including several exons of K_Ca_1.1 was performed using KOD FX Neo DNA polymerase (Toyobo, Osaka, Japan) in a thermal cycler (T100, Bio-Rad Laboratories, Tokyo, Japan). The amplification profile was as follows: a 15-s denaturation step at 96 °C and a 30-s primer extension step at 60 °C. The following PCR primers were used: K_Ca_1.1 (NM_001014797) exon 1–4: 104–960, amplicon = 857 bp; exon 5–14: 860–1849, 990 bp; exon 15–23: 1761–2916, 1156 bp; exon 24–30: 2688–3741, 1054 bp. The amplified products were separated on 1.0% agarose gels, and visualized by ethidium bromide staining. One kbp DNA Ladder One (Nacalai Tesque, Kyoto, Japan) was used as a molecular weight marker.

### 4.3. Measurement of Protein Expression Levels by Western Blotting and Immunocytochemical Staining

Protein lysates were prepared from breast cancer cell lines using RIPA lysis buffer (50 mM Tris-HCl (pH 7.4), 150 mM NaCl, 1 mM ethylenediaminetetraacetic acid (EDTA), 1% Triton X-100, 1% Na-deoxycholate, 0.1% sodium dodecyl sulfate (SDS) with a protease inhibitor mini tablet (Thermo Scientific Pierce, Yokohama, Japan)) for Western blotting, as previously reported [36]. Protein expression levels were measured 72 h after the compound treatment. Equal amounts of protein (20 µg/lane) were subjected to SDS-PAGE (10%). Blots were incubated with anti-VDR (D-6) (Santa Cruz Biotechnology) [56], anti-K_Ca_1.1 (APC-021) (Alomone Labs, Jerusalem, Israel), anti-HDAC2 (H-54) (Santa Cruz Biotechnology) [57], and anti-ACTB (6D1) (Medical & Biological Laboratories (MBL), Nagoya, Japan) antibodies, then incubated with anti-rabbit and anti-mouse horseradish peroxidase-conjugated IgG (Merck Millipore, Darmstadt, Germany), respectively. An enhanced chemiluminescence detection system (GE Healthcare Japan, Tokyo, Japan) was used to detect the bound antibody. The resulting images were analyzed using a VersaDoc5000MP device (Bio-Rad Laboratories, Hercules, CA, USA). The optical density of the K_Ca_1.1 protein band signal relative to that of the ACTB signal was calculated using ImageJ software (Ver. 1.42, National Institute of health (NIH), Bethesda, MD, USA), and protein expression levels in the vehicle control were then expressed as 1.0.

In the immunocytochemical examination, MDA-MB-453 cells were harvested using a sterile cell scraper, and non-permeabilized cells were stained using a rabbit polyclonal K_Ca_1.1 (extracellular) antibody (APC-151, Alomone Labs) plus Alexa Fluor^®^ 488-conjugated goat anti-rabbit IgG secondary antibody (Thermo Fisher Scientific). Stained cells were subjected to an analysis on a FACSCalibur flow cytometer using CellQuest software (BD Biosciences, San Jose, CA, USA) [36].

### 4.4. Measurements of the K_Ca_1.1 Activity by Voltage-Sensitive Dye Imaging and Whole-Cell Patch Clamp Recording

Membrane potential was measured using the fluorescent voltage-sensitive dye DiBAC_4_(3) [58]. Cells were seeded on glass-bottomed tissue culture dishes (Matsunami Grass, Osaka, Japan) and cultured at 37 °C in a 5% CO_2_ humidified incubator with 1 μM calcitriol or calcipotriol for 72 h. Prior to the fluorescence measurements with DiBAC_4_(3), cells were incubated in normal HEPES buffer containing 100 nM DiBAC_4_(3) at room temperature for 20 min. Cells were continuously incubated in 100 nM DiBAC_4_(3) throughout the experiments. The measurement of paxilline-induced depolarization responses were performed in the absence of calcitriol or calcipotriol. In membrane potential imaging, cells loaded with DiBAC_4_(3) were illuminated at a wavelength of 490 nm, and fluorescence images were recorded using an ORCA-Flash2.8 digital camera (Hamamatsu Photonics, Hamamatsu, Japan). Data collection and analyses were performed using an HCImage system (Hamamatsu Photonics). Images were measured every 5 s, and the values of fluorescent intensity (F) were obtained by measuring the average for 1 min (12 images). A whole-cell patch clamp was applied to single MDA-MB-453 cells using the HEKA EPC 10 USB amplifier (HEKA Elektronik, Lambrecht/Pfalz, Germany) at room temperature (23 ± 1 °C). Data acquisition and analysis of whole cell currents were performed using PatchMaster (HEKA Elektronik). The resistance of microelectrodes filled with pipette solution was 3–5 MΩ. Whole-cell currents were measured in voltage-clamp mode and induced by 500 ms voltage steps, every 15 s, from −80 mV to +40 mV at a holding potential of −60 mV. The external solution was (in mM): 137 NaCl, 5.9 KCl, 2.2 KCl, 1.2 MgCl_2_, 14 glucose and 10 HEPES, pH 7.4. The pipette solution was (in mM): 140 KCl, 4 MgCl_2_, 3.16 CaCl_2_, 5 EGTA, 10 HEPES and 2 Na_2_ATP, pH 7.2, with an estimated free Ca^2+^ concentration of 300 nM (pCa 6.5).

### 4.5. Statistical Analysis

The significance of differences among two and multiple groups was evaluated using the Student’s *t*-test and Tukey’s test after the F test and ANOVA, respectively. Significance at *p* < 0.05 and *p* < 0.01 is indicated in the figures. Data are presented as the means ± SEM.

## 5. Conclusions

Our results provide new mechanistic insights into showing that Ca^2+^-activated K^+^ channel K_Ca_1.1 is a new downstream target of VDR signaling and the VDR stimulation enhanced the transcriptional repression of K_Ca_1.1 and its protein degradation in human breast cancer cells. The down-regulation of K_Ca_1.1 is responsible, at least in part, for the antiproliferative effects induced by the VDR stimulation in K_Ca_1.1-positive human breast cancer cells. The molecular mechanisms underlying the down-regulation of K_Ca_1.1 through the VDR signaling pathway in breast cancer cells has yet to be elucidated; however, epigenetic modifications and protein degradation via proteasome pathways represent possible mechanisms.

## Supplementary Materials

Supplementary materials can be found at www.mdpi.com/1422-0067/17/12/2083/s1.
