# Supplementary material for: Down-Regulation of Ca2+-Activated K+ Channel KCa1.1 in Human Breast Cancer MDA-MB-453 Cells Treated with Vitamin D Receptor Agonists
Source: Int J Mol Sci. 2016 Dec 11;17(12):2083. doi: 10.3390/ijms17122083 (PMC5187883; doi:10.3390/ijms17122083)
Supplement: Supplementary file 1 [file ijms-17-02083-s001.pdf]

Supplementary Materials: Down-Regulation of Ca^{2+}-Activated K^+ Channel $K_{Ca}1.1$ in Human Breast Cancer MDA-MB-453 Cells Treated with Vitamin D Receptor Agonists

Anowara Khatun, Mayu Fujimoto, Hiroaki Kito, Satomi Niwa, Takayoshi Suzuki and Susumu Ohya

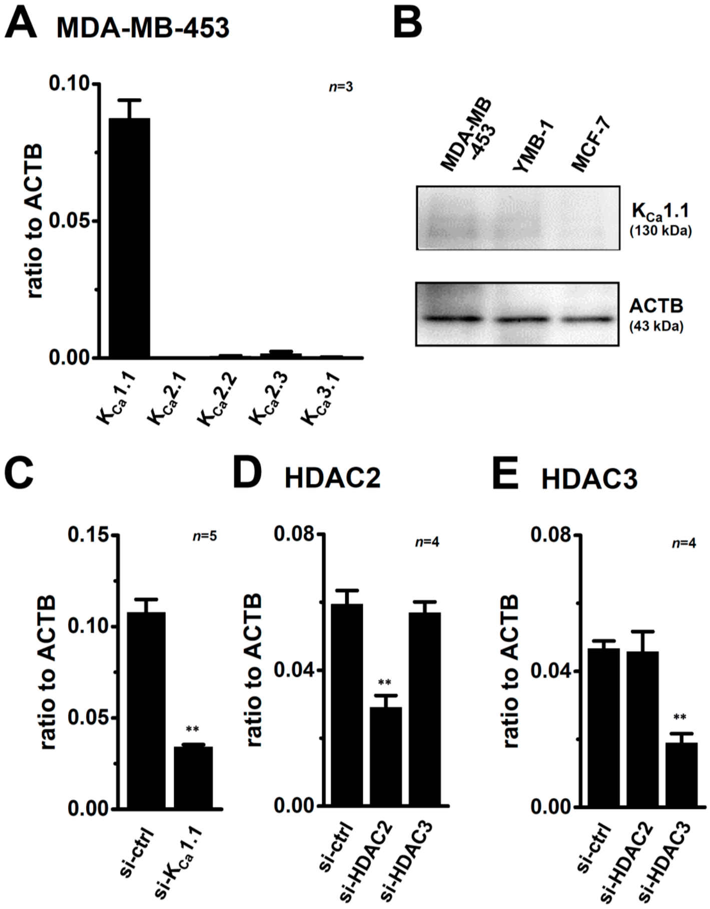

Figure S1. Expression of K_{Ca} channel subtypes in MDA-MB-453 cells, the negative control in Western blotting for $K_{Ca}1.1$, the inhibition of expression levels of $K_{Ca}1.1$/HDAC2/HDAC3 transcripts by the transfection of $K_{Ca}1.1$/HDAC2/HDAC3 siRNA, respectively. (**A**) Real-time PCR assays for $K_{Ca}1.1$, $K_{Ca}2.1$, $K_{Ca}2.2$, $K_{Ca}2.3$, and $K_{Ca}3.1$ in MDA-MB-453 cells (n = 3 for each). Expression levels were expressed as a ratio to ACTB; (**B**) Protein lysates of MDA-MB-453, YMB-1, and MCF-7 cells were probed by immunoblotting with anti-$K_{Ca}1.1$ (upper panel) pretreated with excess antigens and anti-ACTB (lower panel) antibodies on the same filter; (**C**) Real-time PCR assay for $K_{Ca}1.1$ in MDA-MB-453 cells transfected with control siRNA (si-ctrl) and $K_{Ca}1.1$ siRNA (si-$K_{Ca}1.1$) (n = 5 for each); (**D**,**E**) Real-time PCR assay for HDAC2 (**D**) and HDAC3 (**E**) in MDA-MB-453 cells transfected with control siRNA (si-ctrl), HDAC2 siRNA (si-HDAC2), and HDAC3 siRNA (si-HDAC3) (n = 4 for each). Results are expressed as means ± SEM. **: $p < 0.01$ vs. si-ctrl.

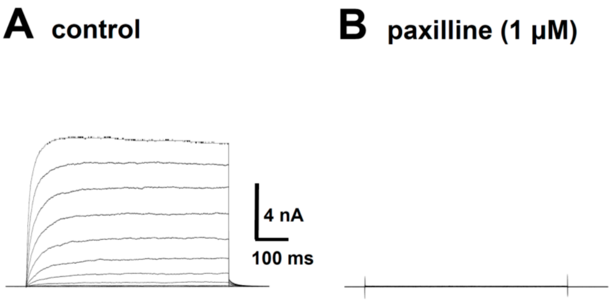

Figure S2. Effects of 1 μM paxilline on outward K^+ currents in MDA-MB-453 cells. Currents were elicited by depolarizing voltage-step to +40 mV from holding potential (−60 mV) with 10 mV increment (**A**); The currents were almost completely inhibited by application of 1 μM paxilline (**B**).

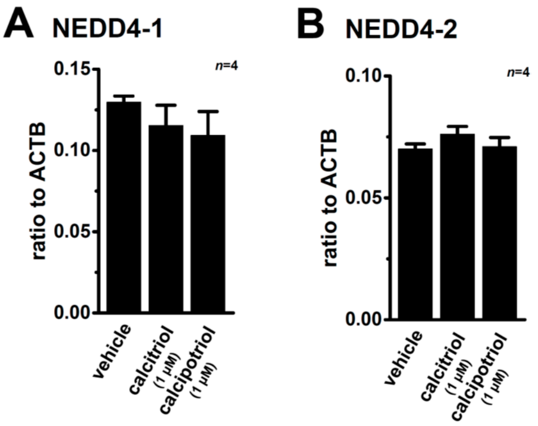

Figure S3. Effects of treatment with VDR agonists on transcriptional expression levels of E3 ubiquitin-protein ligases (NEDD4-1 and 4-2) in MDA-MB-453 cells. (**A**,**B**) Real-time PCR assay for NEDD4-1 (**A**), and NEDD4-2 (**B**) in VD agonist-treated MDA-MB-453 (n = 4 for each). Expression levels were expressed as a ratio to ACTB. Results are expressed as means ± SEM.
